# Anxiety is associated with extraneous cognitive load during teaching using high-fidelity clinical simulation

**DOI:** 10.1080/10872981.2021.1994691

**Published:** 2021-10-28

**Authors:** Salim Fredericks, Mostafa ElSayed, Mustafa Hammad, Omneya Abumiddain, Leila Istwani, Abdulla Rabeea, Fiza Rashid-Doubell, Abdelhaleem M.E. Bella

**Affiliations:** Rsci (Bahrain). School of Medicine, Royal College of Surgeons in Ireland, Medical University of Bahrain, Adliya, Bahrain

**Keywords:** Simulation, cognitive load, anxiety, state-trait anxiety inventory, medical education

## Abstract

High-fidelity clinical simulation is currently a well-established teaching tool. However, high-fidelity representations of patients in critical conditions have the potential to elicit emotions among learners and impact their cognitive load (CL). Teaching with clinical simulation may induce both emotional and cognitive overloads. The relationship between anxiety and CL during clinical simulation was studied. Forty-one undergraduate medical students participated in this study; 19 males and 22 females. The state-anxiety component of State-Trait Anxiety Inventory was administered during clinical simulation teaching sessions at time points: pre-scenario, post-scenario and post-debriefing. The Cognitive Load Scale (Leppink et al.) questionnaire was also completed post-scenario. This assessed the three components of CL: intrinsic cognitive load (ICL), extraneous cognitive load (ECL) and self-perceived learning (SPL). Median CL scores for ICL, ECL and SPL were compared between groups of low-anxiety and high-anxiety participants using a Mann-Whitney U test. State-anxiety scores were high for both the pre-scenario and post-scenario time points with a significant reduction following post-debriefing. The median (interquartile range) state-anxiety scores were 41.0 (33.0–50.0), 46.0 (33.0–52.0) and 31.0 (23.0–39.0) for the pre-scenario, post-scenario and post-debriefing time points respectively. Students with high state-anxiety had higher ECL scores (median = 2.0) than students with low state-anxiety (median = 0.9) at the post scenario time point (U = 220, p = 0.043). No statistical relation was seen with state-anxiety for either ICL or SPL. State-anxiety immediately after the simulation scenario is associated with ECL but not ICL or SPL.

## Introduction

Currently, clinical simulation is well-established as an effective teaching tool for use with medical students and for the training of qualified professionals [1,2]. The acquisition of clinical skills has become the most common learning objective for simulation in medical education[1]. Complex clinical scenarios such as team-responses to emergencies may be simulated with life-like computer-controlled mannequins. Modern mannequins are sophisticated in their design and generate real-time changes in haemodynamics, blood gases, and vital signs in response to clinical interventions. This allows for simulated interventions to be performed that are deemed too dangerous or too complex to practise on actual patients. The use of high-fidelity simulation, therefore, seems appropriate for teaching compound clinical competencies in a safe, supported, and controlled manner. However, the very nature and the realism of clinical simulations have both favourable and unfavourable consequences. Although the educational benefits of clinical simulations are clear, these high-fidelity representations of human frailties and critically ill patients can elicit negative emotions in learners [3–5]. These emotions may adversely impact the process of learning[6]. Clinical simulation teaching sessions, as with many other novel learning environments, induce anxiety in learners[7]. High levels of anxiety are detrimental to learning as they overload learning processes [8,9]. In contrast, low levels of anxiety provide insufficient stimuli to motivate individuals to learn. Thus, boredom and lack of engagement may be associated with low anxiety. Therefore, a precarious balance exists between optimal learning and anxiety levels during teaching using simulation[5].

Detrimental effects of anxiety also impact cognitive capacity, with high anxiety being associated with low performance during problem-solving[10]. The more authentic the simulation and the more dynamic the interactions within the simulation setting, the greater the potential for cognitive overload. The finite capacity of the *working memory* of learners with high anxiety levels becomes deluged with the excessive superfluous thoughts generated through anxiety. This notion of *working memory* is central to the conceptual framework of cognitive load theory (CLT), which has been used to describe how instructional design may influence effective learning[11]. The CLT framework is considered appropriate for designing teaching activities involving simulation[12]. CLT assumes that working memory capacity is fixed and that learning is hindered when this memory capacity is overloaded. This overloading inhibits the process of transferring information from the narrow realm of *working memory* to the more expansive *long-term* memory [11,13]. The CLT framework is based on the premise that three components contribute to the total cognitive load (CL) placed upon the learner[13]. These three components being: the *intrinsic* cognitive load (ICL) [14] of the learning task itself, the *extraneous* cognitive load (ECL) of the actual learning situation, and the germane cognitive load [14] or *self-perceived learning* (SPL)[15]. Intrinsic and extraneous CL are widely accepted factors of the CL framework, whilst germane is more controversial [16,17]. Germane cognitive load has been reinterpreted as ‘self-perceived learning’ [15,18]; i.e., the individual’s perception that learning has taken place as assessed by one particular subjective measure of CL (Leppink et al.; 2013) [19,20]. High scores on the SPL scale have been reported to be predictive of high academic performance[15]. However, few published studies report upon the components of CL in relation to simulation. Research has focused on measures of total CL[21]. Studies have shown that high CL during training with clinical simulation is associated with decreased learning and impaired performance of clinical skills [22,23]. Further, emotions experienced during training with simulation are associated with total CL ratings and poorer learning outcomes are associated with higher total CL scores [24,25].

This theoretical understanding of the components of memory overload offers the practical application of being a guide for instructional design and helps explain how learning a new complex task taxes the learner’s cognitive system[13]. Drawing upon the two concepts of intrinsic and extraneous CL is particularly helpful in evaluating learning activities and learning environments [12,26]. The *intrinsic load* of a simulation teaching session is the inherent difficulty imposed upon a learner within the learning task and the complexity of the simulation scenario itself. Learners that are new to a task are more prone to experience cognitive overload. The learner’s prior knowledge and experience are the primary factors affecting ICL, with emotional states having minimal influence.

In contrast, emotional states such as anxiety would be expected to affect the *extraneous load*[27], i.e., the working memory consumed by mental processes imposed by instructional or context elements that are neither necessary nor appropriate for learning. Clinical environments contain several elements that trigger specific cognitive interactions between the individuals involved and the general setting. Simulations faithfully replicate these same elements and interactions; although, they may not be core to the learning objectives. Thus, the scenario, the relevant clinal data, the associated assessments and the instructions provided all contribute to the consumption of the working memory capacity. Moreover, the high-fidelity representations of life or death scenarios influence emotional states, affecting CL and impacting learning [4,28–30]. Studies relating emotional states to CL have suggested that emotions are associated with ECL rather than ICL. Fraser et al. demonstrated that exposing learners to the unexpected death of a simulated patient increased total CL and had poorer learning outcomes. The authors suggested that this relationship between emotional and total cognitive loads stems from an extraneous overload[24]. Pawar et al. investigated the impacts of simulation on emotional states and CL in an intensive care setting. They expressly avoided the unexpected death of a critically ill patient within the scenario in order to reduce ECL. They reported no correlation between total CL and the various emotional descriptors investigated[21]. It has been argued that both positive and negative emotions can lower the capacity of working memory by generating an extraneous CL; triggered by task-irrelevant processing[25]. Thus, both cognitive overload and situational anxiety are potential barriers to effective learning. Therefore, there is a need to understand cognitive overload in relation to high-anxiety prone learners within the context of clinical simulation classes in order to develop suitable and targeted protocols to enhance their learning.

The aim of this study was to assess the effect of participating in clinical simulation on subjective measures of anxiety and CL with final year undergraduate medical students. Further, the relationship between measured anxiety scores and the three components, ICL, ECL and SPL, was examined. The hypothesis of the present study was that high levels of anxiety during the enactment of the scenario segment of simulation sessions are associated with an *extraneous* overload in learners.

## Methods

### Study design and participants

This cross-sectional study was conducted in the ANON. State-anxiety, trait-anxiety, and CL were assessed at three distinct time points during clinical simulation teaching sessions. Participants were recruited from students enrolled on a five-year undergraduate medical programme structured with an integrated curriculum. All students in their fifth and final year (semester 10) were invited to participate. Participation in this study was voluntary and written informed consent was collected from all participants before enrolment onto the study. The study was approved by the Research Ethics Committee of ANON.

### Clinical simulation teaching sessions

Teaching sessions using simulation took place over the period from January to April 2019. Data were collected over nine teaching sessions which were scheduled to last for 90 minutes. Each clinical teaching event had a group of 5–8 students and three facilitators. All students entered the scenario as a group and played the role of interns. Generally, the scenarios aimed to provide students with experiential training in inpatient competencies relevant to caring for hospitalised patients. More specifically students were expected to work as a group to gather relevant clinical information to obtain a focussed patient history and a collateral patient history, examine the simulated patient, order and interpret the results of investigations, commence appropriate treatment and communicate effectively with the patient, staff and patient’s family members. One student was always appointed as the team-leader for each session. Students had not had previous exposure to teaching with simulation in earlier years on the course.

The three facilitator and two actors followed pre-determined scripts during the scenario. The same facilitators and actors were involved in all sessions. The fascinators played the roles of a nurse and two senior medical residents. The nurse was available in the simulation theatre, and medical staff were available via the telephone. The patient and the patient’s family members were voiced by actors. The scenarios were run in an Emergency Bay in a purpose-built patient simulation centre. The level of the simulation fidelity was considered high[31], it had strong components of equipment fidelity, environmental fidelity, and psychological fidelity[32]. All sessions were conducted using the same human patient simulator (HAL® S3101, Gaumard Scientific, FL, USA). Medications and equipment required were available in the simulated clinical environment. Necessary imaging was provided digitally in the simulated setting. All scenarios were video recorded for later use during debriefing.

Each teaching session comprised of three segments: briefing, scenario, and debriefing. The *briefing* segment (approximately 10 minutes) focused on introducing the students to the physical environment including the mannequin. Discussions of the expected learning outcomes and objectives of the session were also outlined. The *scenario* segment (approximately 15 minutes) involved a very brief description of the scenario. The students were then asked to manage the case while supported by their facilitators. During the *debriefing* segment (approximately 45 minutes) the students reflected upon their thoughts and actions during the simulation session. Principles of ‘debriefing with good judgment’ were adopted[33]. No formal assessments were included. Feelings regarding their behaviours and decisions made were explored during debriefing. Issues such as history taking, examination, investigation and management, team dynamics, and communication were discussed. A review of the video recording of the enacted scenario was used as an adjunct to oral feedback during debriefing. The senior facilitator lead all the briefing sessions.

### Scenarios

There were two distinct scenarios delivered on two separate occasions. One scenario involved managing a medical emergency and the other a surgical emergency. The first session to which students were exposed, either medical or surgical, was dependent on block-randomisation using the class list and the timetabling procedure.

The medical scenario was titled *infective exacerbation of chronic obstructive pulmonary disease (COPD)*. The scenario involved a 68-year-old underweight Caucasian male. The students in their roles as interns were asked to see this patient who presents to the Emergency Department with a three-day history of a worsening productive cough, wheeze, and dyspnoea. His temperature was 38.5°C, BP: 122/78, HR: 98 RR: 24 and O_2_ saturation of 87% on room air. He had two previous presentations with the same complaint over the last six months. He had a history of smoking (40 pack years, and he quit two years ago). He lived alone and his daughter lived nearby. His wife had died a year earlier. His was diagnosed with infective exacerbation of COPD, complicated by type II respiratory failure.

The surgical scenario *small bowel obstruction* involved a 75-year-old male who complained of abdominal pain and vomiting. At the time of presentation, he was three days post-resection of a small bowel lymphoma with primary anastomosis. He had a history of prostate cancer (successfully treated 10 years ago) and cholecystectomy (five years earlier). He had clinical signs of dehydration including dry mucus membranes and tachycardia. His abdomen is distended with diffuse tenderness and hyperactive bowel sounds. His temperature was 37°C, BP: 135/85, HR: 120 RR: 18 and O_2_ saturation of 99% on room air. At the time he was married with adult children. He was diagnosed with small bowel obstruction based upon PFA and CT scan imaging.

### Data collection methods

**Figure 1.**
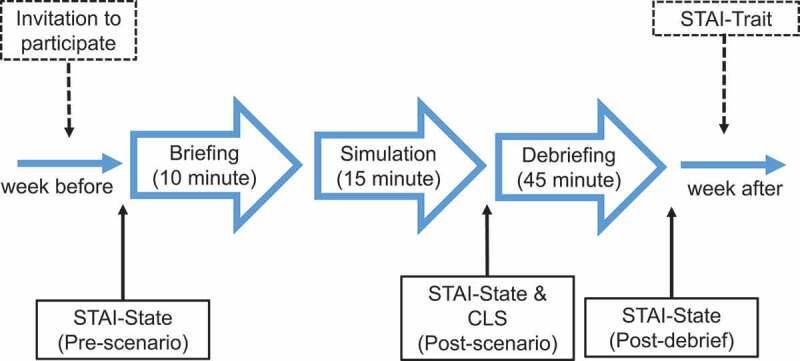
Data collection time-points in relation to the structure of each clinical simulation teaching session. STAI: State-Trait Anxiety Inventory, CLS: cognitive load scale

Questionnaires were used to assess state-anxiety, trait-anxiety, and CL. Each participant had his or her state-anxiety measured at three time points: before the initial briefing (pre-scenario), after the management of the scenario (post-scenario), and once again after the debriefing (post-debriefing). A self-administered questionnaire that measured CL was completed following the scenario (post-scenario). Trait-anxiety was assessed only once for each study participant. This was on a separate day usually one week after the scheduled teaching session, this was to reduce the number of forms to be filled out by the participants on the day of the study. These time points and data collections are summarised in Figure 1.

### State-Trait Anxiety Inventory (STAI)

The State Trait-Anxiety Inventory-Trait (STAI-T) was used to assess the each participant’s natural tendency to be anxious (trait-anxiety) one week after the simulation-teaching session[34]. The State Trait-Anxiety Inventory-State (STAI-S) was used to assess each participant’s transitory emotional state caused by the situation (state-anxiety) at three time points across the teaching session[34]. It was administered once again following the debriefing session when the whole teaching activity was completed. STAI-S consisted of 20 items with a four-point Likert scale for each item. Thus, scores for both trait-anxiety and state-anxiety ranged between 20 and 80 where 20 indicated a low level of anxiety and 80 indicated a high level. The time for completion of the questionnaires varied from three to six minutes. Calculations were based on the scores generated for individual questionnaires. In addition, scores were dichotomised into descriptions of anxiety levels i.e., ‘low-anxiety’ or ‘high anxiety’. High trait-anxiety was defined by a STAI-T score ≥40 and high state-anxiety was defined by a STAI-S score ≥36. These two anxiety cut-off values were based on scores greater than the mean baseline scores for college students as reported in the STAI manual[35]. Several research reports have used the same cut-off values [28,36]. The reported internal consistency of the STAI is high, with Cronbach’s alpha values of 0.92 for both STAI-T and STAI-S[35].

### Cognitive load scale

CL was measured using a subjective scale. The cognitive load scale (CLS) is a 13-item inventory that was developed and validated by Leppink and et al [19], and written permission was obtained from its developer by e-mail prior to the study. The CLS used a 10-point semantic rating scale ranging from ‘not at all the case’ to ‘completely the case’. CLS measured participant’s subjective ratings of the listed 13 items: items 1 to 4 measured the ICL; items 5 to 8 measured the ECL; and the items 9 to 13 measured the students’ *self-perceived learning* (SPL) formally referred to as germane cognitive load[15]. Examples of items from CLS survey included: ‘The content of the activity was very complex.’, ‘I invested a very high mental effort in unclear and ineffective explanations and instructions in this activity’, and ‘This activity really enhanced my understanding of the content that was covered’, which are representative items from the ICL, ECL and SPL sections respectively. The internal consistency of the three components of CL is reported to be high, with Cronbach’s alpha values of 0.81, 0.75 and 0.82 for ICL, ECL and SPL respectively[19]. Participants were asked to return completed CLS paper forms to the researchers immediately after the clinical simulation component of the teaching session. The time for completion of the questionnaire varied between three to six minutes.

### Data analyses

Non-parametric methods were used to analyse the data. Data were expressed as median and interquartile ranges for continuous variables and as number and percentage for categorical variables. Chi-squared or Fisher’s exact tests were performed to compare the frequencies. Friedman tests were performed to compare median scores used for assessing the levels of self-reported state-anxiety (STAI-S) at the pre-scenario, post-scenario, and post-debriefing time points. Mann-Whitney U-tests were performed to compare median anxiety scores between gender, and medical versus surgical scenarios. The normality of distribution of results was determined using a Sharipo-Wilk test. To assess the relationship between CL rating and anxiety scores Pearson’s correlation coefficients were used. P values < 0.05 were considered statistically significant. The reliability of STAI and CLS tools was assessed using Cronbach’s alpha coefficient with a value of 0.7 or above considered as indicative of acceptable reliability[37]. All statistical tests were carried out using IBM SPSS Statistics 25.

## Results

Forty-five students were enrolled on the compulsory clinical simulation classes during the period in which the study was carried out. Forty-one of the 45 of the students agreed to participate. There were 19 male and 22 female student participants. The median age of the students was 23 (IQR: 22–24). Data were collected for STAI and CLS surveys. Initial analysis of the results showed that neither STAI scores (Sharipo-Wilk, n = 59, p < 0.01) nor ECL scores (n = 59, p < 0.01), were normally distributed, so further analysis used non-parametric methodology.

State-anxiety scores were high for both the pre- scenario and post-scenario time points with a significant fall following debriefing. The median STAI-S scores were 41.0 (IQR: 33.0–50.0), 46.0 (IQR: 33.0–52.0) and 31.0 (IQR: 23.0–39.0) for the pre-scenario, post-scenario, and post-debriefing time points, respectively. State-anxiety was measured across all three time points of pre-scenario, post-scenario, and post-debriefing on a total of 59 occasions. This included a maximum of two sessions (the medical scenario and the surgical scenario) for each student. A Friedman test was carried out to compare the STAI-S scores across the three time points. There was found to be a significant difference between the scores χ^2^_(2)_ = 45.313, p < 0.001. Dunn-Bonferroni post hoc tests were carried out and there were significant differences between the pre-scenario and post-debriefing (p < 0.001) as well as the post-scenario and post-debriefing (p < 0.001) after Bonferroni adjustments. There were no significant differences between pre-scenario and post-scenario.

The average STAI-S scores were significantly higher for females when compared to males. Three Mann-Whitney U tests showed that female students had higher STAI-S scores than male students (p < 0.05) for the pre-scenario, post-scenario, and post-debriefing collection time points. Similarly, there were significant differences in these state-anxiety scores for those participants with low or high trait-anxiety scores. Three Mann-Whitney U tests also showed that students with high trait-anxiety had higher STAI-S scores (p < 0.001) when compared to students with low trait-anxiety for the three time points. Two teaching sessions were conducted, and these teaching slots were randomised as either medical or surgical scenarios for the first or second session. A Mann-Whitney U test showed that students with their first exposure to simulation (Mdn = 48) had higher STAI-S scores when compared to students on their second exposure to simulation for that academic year (Mdn = 37) at the post-scenario time point only (U = 315.5, p = 0.017). These data are summarised on Table 1. No statistically significant differences were seen for trait-anxiety between any of the groups (i.e., male v’s female, leaders v’s non-leaders, first v’s second exposure to teaching with simulation and medical v’s surgery scenarios).

**Table 1.** Median (interquartile range) STAI-state scores for demographic data across the three data collection points. Friedman test was used to compare the STAI-state scores across the three time points. Significant differences were seen between; male v female, low v high trait-anxiety, 1^st^ v 2^nd^ exposure to simulation and medicine v surgery scenarios as indicated: ^†^ p < 0.05 and ^‡^ p < 0.01

	N	Pre-scenario	Post-scenario	Post-debrief	Friedman test
All	59	41 (33–50)	46 (33–52)	31 (23–39)	*χ*^[2]^= 45.313, p < 0.001
Male	27	35 (27–49) ^‡^	37 (26–48) ^†^	27 (22–35) ^†^	*χ*^[2]^= 24.263, p < 0.001
Female	32	45 (39–51)	48 (38–57)	34 (27–40)	*χ*^[2]^= 22.563, p < 0.001
Low trait-anxiety	26	32 (26–35) ^‡^	37 (25–48) ^‡^	28 (22–32) ^‡^	*χ*^[2]^= 16.227, p < 0.001
High trait-anxiety	33	49 (42–53)	48 (39–57)	35 (27–43)	*χ*^[2]^= 32.508, p < 0.001
Non leader	51	41 (33–52)	47 (34–53)	32 (25–39)	*χ*^[2]^= 36.437, p < 0.001
Leader	8	41 (33–48)	39 (21–48)	23 (20–35)	*χ*^[2]^= 9.867, p = 0.007
1^st^ exposure	18	42 (36–52)	48 (41–59) ^†^	32 (26–41)	*χ*^[2]^= 15.114, p = 0.001
2^nd^ exposure	21	40 (32–53)	37 (25–50)	29 (20–38)	*χ*^[2]^= 20.632, p < 0.001
Medicine	30	46 (35–53) ^†^	45 (37–56)	32 (23–42)	*χ*^[2]^= 28.394, p < 0.001
Surgery	29	38 (31–44)	47 (28–51)	31 (22–37)	*χ*^[2]^= 20.685, p < 0.001

High trait-anxiety was associated with high ECL scores. Post-scenario STAI-S scores and ECL rating were found to be positively correlated (r = 0.356, p < 0.010). Students with high state-anxiety had higher ECL scores (Mdn = 2.0) than students with low state-anxiety (Mdn = 0.9) at the post scenario time point, as tested using a Mann-Whitney U test (U = 220.0, p = 0.034). No significant correlations were found for either ICL or SPL with any of the STAI scores. CLS are summarised in relation to anxiety scores on Table 2.

**Table 2.** Median (interquartile range) rating for: intrinsic cognitive load (ICL); extraneous cognitive load (ECL); and self-perceived learning (SPL) as related to low and high anxiety groups. Anxiety was assessed using State Trait Anxiety Inventory – Trait (STAI-T) and State Trait Anxiety Inventory – State (STAI-S) at pre-scenario, post- scenario, and post-debriefing time points. Mann-Whitney U test was used to compare median cognitive load rating from low and high anxiety groups

	STAI-T		STAI-S pre-scenario		STAI-S post-scenario		STAI-S post-debriefing	
	low	high	low	high	low	high	low	high
	(n = 27)	(n = 32)	(n = 21)	(n = 38)	(n = 16)	(n = 43)	(n = 41)	(n = 18)
ICL	4.2 (2.0–5.7)	3.7 (2.7–5.0)	4.2 (2.4–5.7)	3.7 (2.5–4.7)	3.7 (1.6–5.2)	3.7 (2.7–5.5)	3.7 (2.5–5.5)	4.2 (2.5–5.5)
	U = 417.5, p = 0.677		U = 354.5, p = 0.393		U = 257.0, p = 0.239		U = 374.5, p = 0.702	
ECL	2.0 (0.7–2.7)	1.0 (0.5–3.0)	2.0 (0.6–2.9)	1.0 (0.5–3.0)	0.9 (0.6–1.9)	2.0 (0.7–3.0)	1.6 (0.5–2.7)	1.5 (1.0–3.5)
	U = 391.0, p = 0.417		U = 355.0, p = 0.397		U = 220.0, p = 0.034		U = 323.5, p = 0.238	
SPL	6.0 (4.2–8.0)	6.2 (5.1–8.0)	6.8 (4.2–8.1)	6.0 (5.0–7.8)	6.6 (4.5–8.1)	6.0 (4.8–8.9)	6.6 (4.7–8.2)	6.0 (5.0–7.0)
	U = 401.5, p = 0.513		U = 375.5, p = 0.598		U = 302.5, p = 0.479		U = 323.5, p = 0.239	

The Cronbach’s alpha coefficient was used to assess the reliability of the composite scores generated by the questionnaires employed. STAI-S consisted of 20 items (Alpha = 0.93) and the STAI-T also consisted of 20 items (Alpha = 0.91). The CL questionnaire consisted of 13 items with three subscales. ICL subscale consisted of 4 items (Alpha = 0.84), ECL subscale consisted of 4 items (Alpha = 0. 70), and SPL subscale consisted of 5 items (Alpha = 0.94).

## Discussion

This study aimed to assess the effect of participation in teaching with simulation on anxiety, ICL, ECL and SPL. A further aim was to compare participants’ anxiety ratings with their ICL, ECL, and SPL ratings. State-anxiety ratings were high for both the pre- and post-scenario time points, with a reduction after debriefing, suggesting an association between simulation and anxiety. Students with high state-anxiety had higher ECL ratings at the post scenario time point than students with low state-anxiety. This correlation suggests that state-anxiety during simulation is associated with ECL. In contrast, there were no statistical relationships between state-anxiety and ICL or SPL, suggesting that state-anxiety is neither associated with ICL nor SPL.

The post-scenario increase in anxiety as described here may impact learning[10]. Previous studies have shown that emotional states affect measures of cognitive load at the post-scenario stage of training with simulation [21,24]. However, these studies focused on total CL rather than ICL and ECL components. Young and Sewell have commented that several factors, internal to the learner, may contribute to extraneous load during teaching with simulation. These factors include: learner’s self-consciousness at being observed, fatigue, internal thoughts and distractions and also anxiety[27]. Therefore, the focus of the current study was to examine both ICL and ECL in relation to one of these factors that are internal to the learner, anxiety. ECL may be influenced by the generation of superfluous thoughts that are associated with anxiety. Our finding supports this notion that emotional load is associated with extraneous CL, specifically, and that state-anxiety correlates with ECL but not ICL or SPL. Dissimilar to ECL, ICL is highly dependent on prior knowledge and experience and not on the learner’s emotional state. It is widely accepted that the optimal design for teaching with simulation should minimise ECL [11,13,21]. Measures taken to reduce anxiety during simulations may help to minimise ECL.

The observed peak in state-anxiety followed by a fall after debriefing suggests that enacting the simulation scenario may have an anxiogenic effect whilst debriefing reduces anxiety. Participants with high trait-anxiety had high state-anxiety scores across the three data collection time points: i.e., pre-scenario, post-scenario and post-debriefing. These findings are in agreement with those of Evain et al., who also showed a reduction in state-anxiety scores after debriefing and that participants with anxious personalities were at risk of having high state-anxiety even after the debriefing[38]. The calming effect of debriefing may be related to the reflective bidirectional discussions during this segment of the teaching session [2,39]. Effective debriefing may allow students experiencing high anxiety during the scenario to attain a successful learning experience.

This study has certain limitations. The first limitation was the small sample size. The study was conducted in a small medical school, and the number of participants could not be increased for a student year group. There was no statistical difference in STAI-S scores between leaders and non-leaders. However, there were few sessions, and therefore the number of participants who were leaders was low. We could not draw firm conclusions about the role of group leaders concerning state-anxiety or CL. Moreover, the singling out of leaders in each scenario may be an important confounder that could not be easily controlled for in this setting. Increasing the sample size may have elucidated important issues related to the appointed leaders. Secondly, variability would have existed among the facilitators and the debriefers. Although the same facilitators and debriefers conducted each of the sessions and standardised scripts were employed, the nature of this form of teaching means that the student responses and reactions would be unique and specific for each simulation. There would be inevitable variations in the debriefing discussions and slight deviations from scripts in the scenarios. This variability may directly or indirectly affect variation in measured anxiety and CL scores. Thirdly, we did not include any measures of effective learning. Objective assessments of performance outcomes may have better elucidated the relationship between performance, CL, and anxiety. It has been shown that highly anxious individuals perform better than individuals with low-anxiety levels in non-stressful situations[9]. Neither cognitive nor emotional load during teaching activities translate into performance in the clinic. Lastly, this study was conducted in a single higher educational institution. Therefore, the findings may not necessarily be generalisable to other settings.

## Conclusions

Debriefing reduces state-anxiety associated with clinical simulation. High state-anxiety, immediately after the simulation scenario is associated with *extraneous* but not *intrinsic* CL or self-perceived learning.
